# Supplementary material for: Reusable Nano-Zirconia-Catalyzed Synthesis of Benzimidazoles and Their Antibacterial and Antifungal Activities
Source: Molecules. 2021 Jul 12;26(14):4219. doi: 10.3390/molecules26144219 (PMC8304051; doi:10.3390/molecules26144219)
Supplement: Supplementary file 1 [file molecules-26-04219-s001.zip › molecules-1286748-supplementary.pdf]

Supporting Information

Reusable Nano- Zirconia- Catalyzed Synthesis of Benzimidazoles and Their Antibacterial and Antifungal Activities

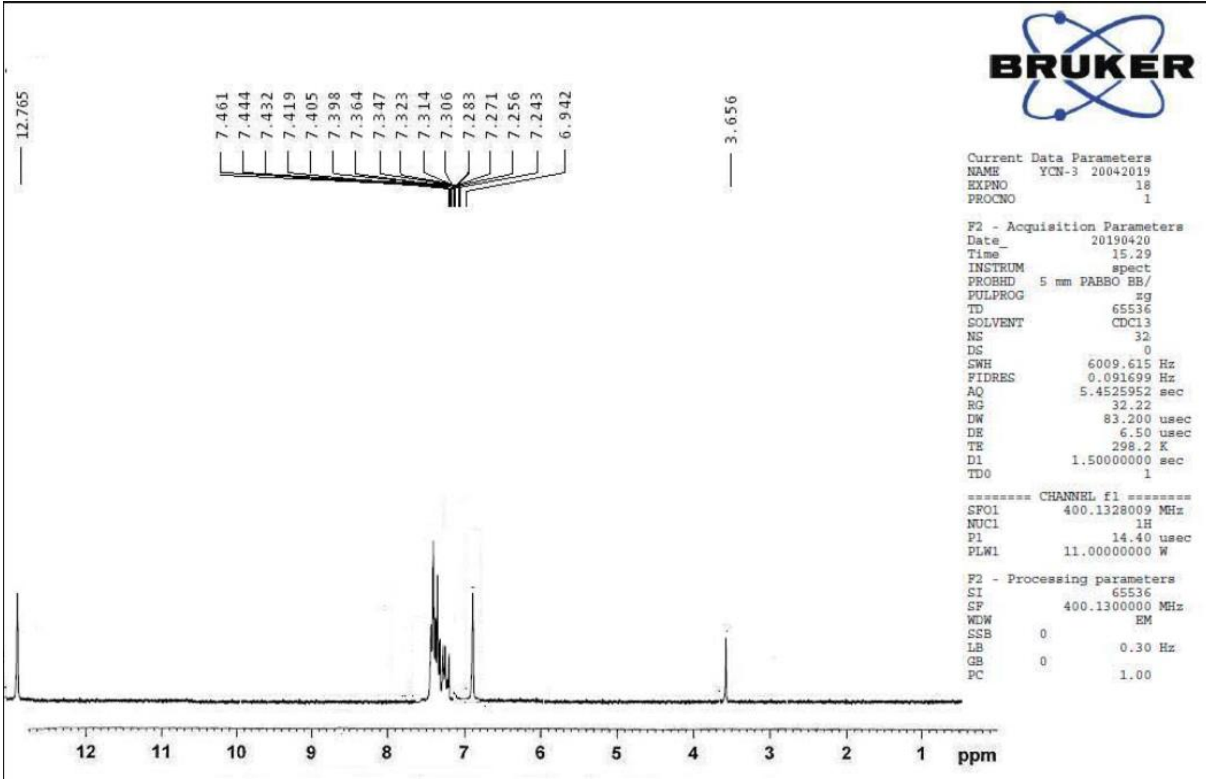

2-(2-iodo-3, 5-dimethoxyphenyl)-1H-benzimidazole (3j)

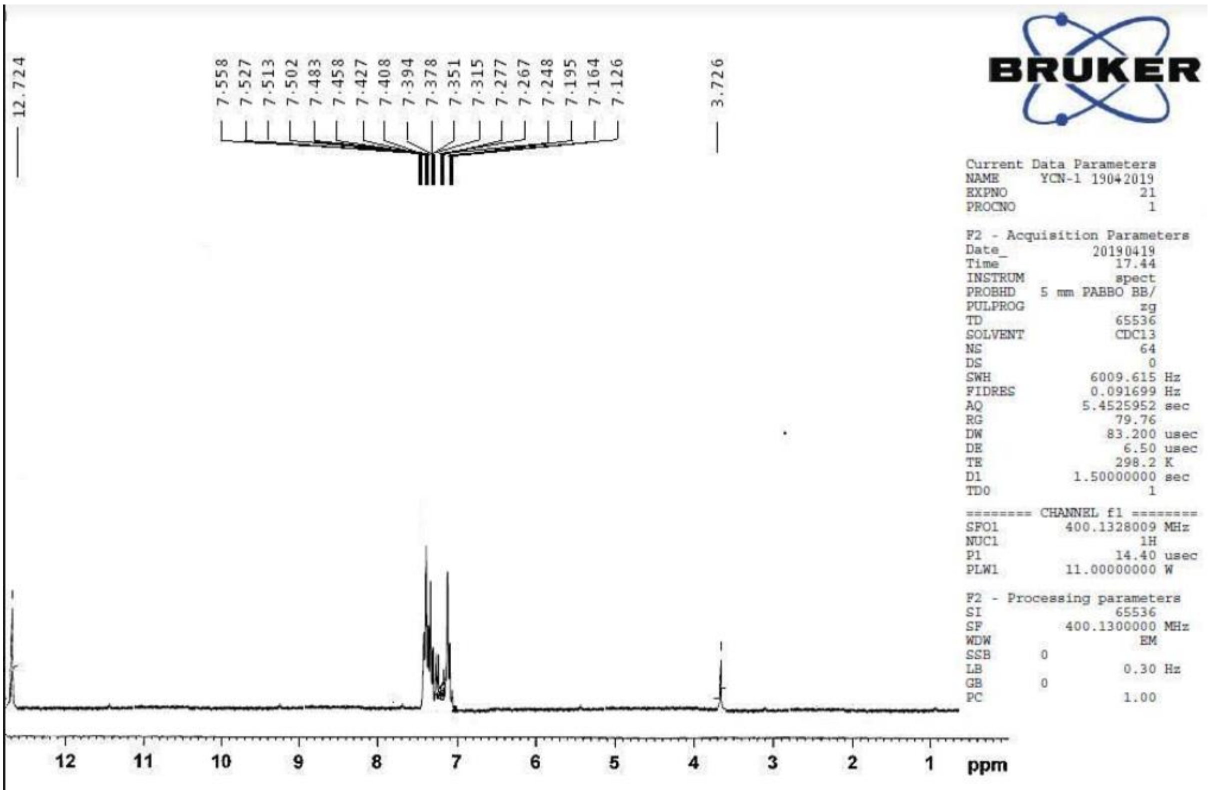

2-(2-bromo-3,4-dimethoxyphenyl)-1H-benzimidazole(3i):

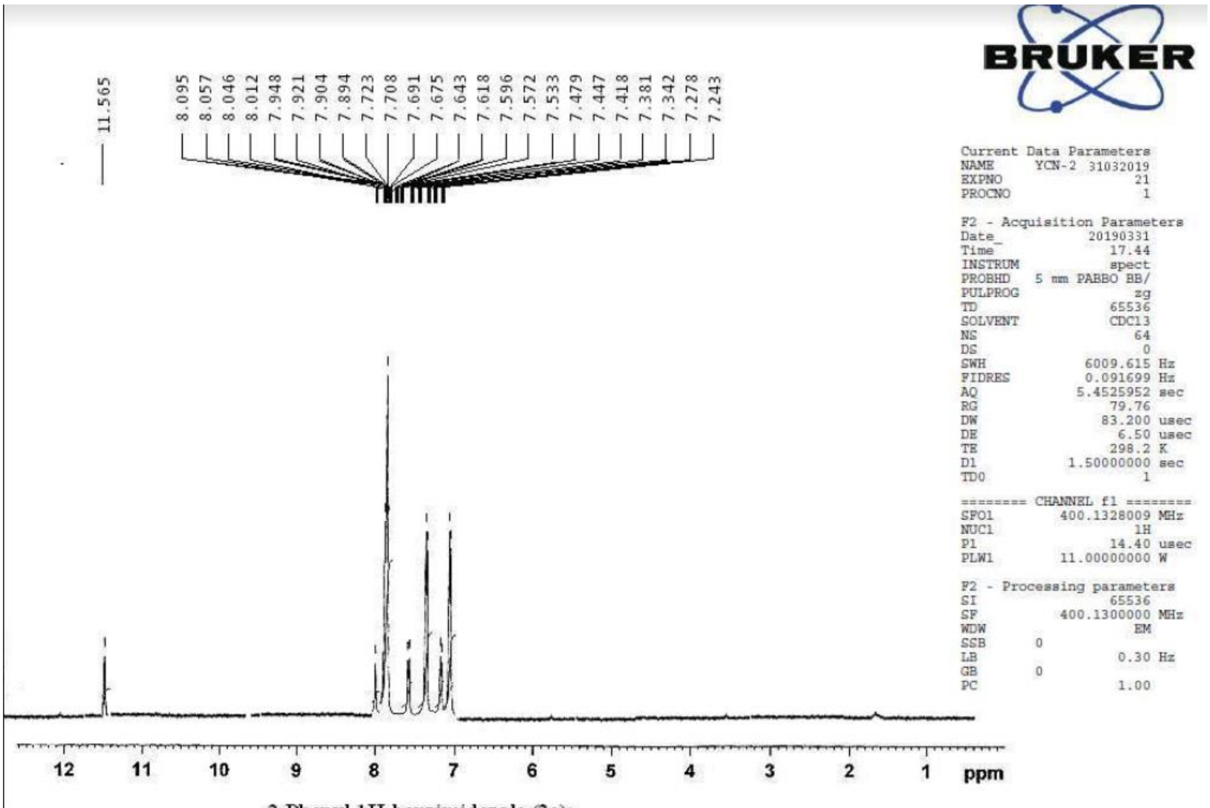

2-Phenyl-1H-benzimidazole (3a):

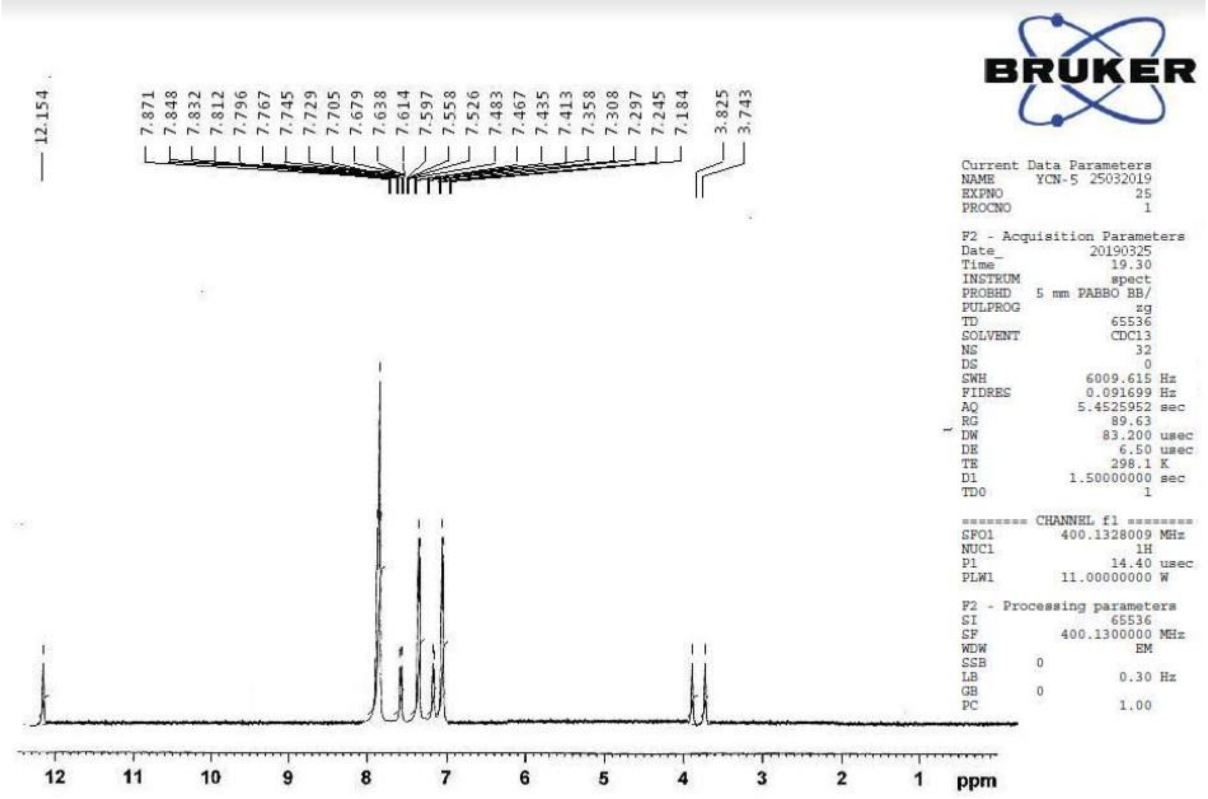

2-(3, 5-Dimethoxyphenylhenyl)-1H-benzimidazole (3c):

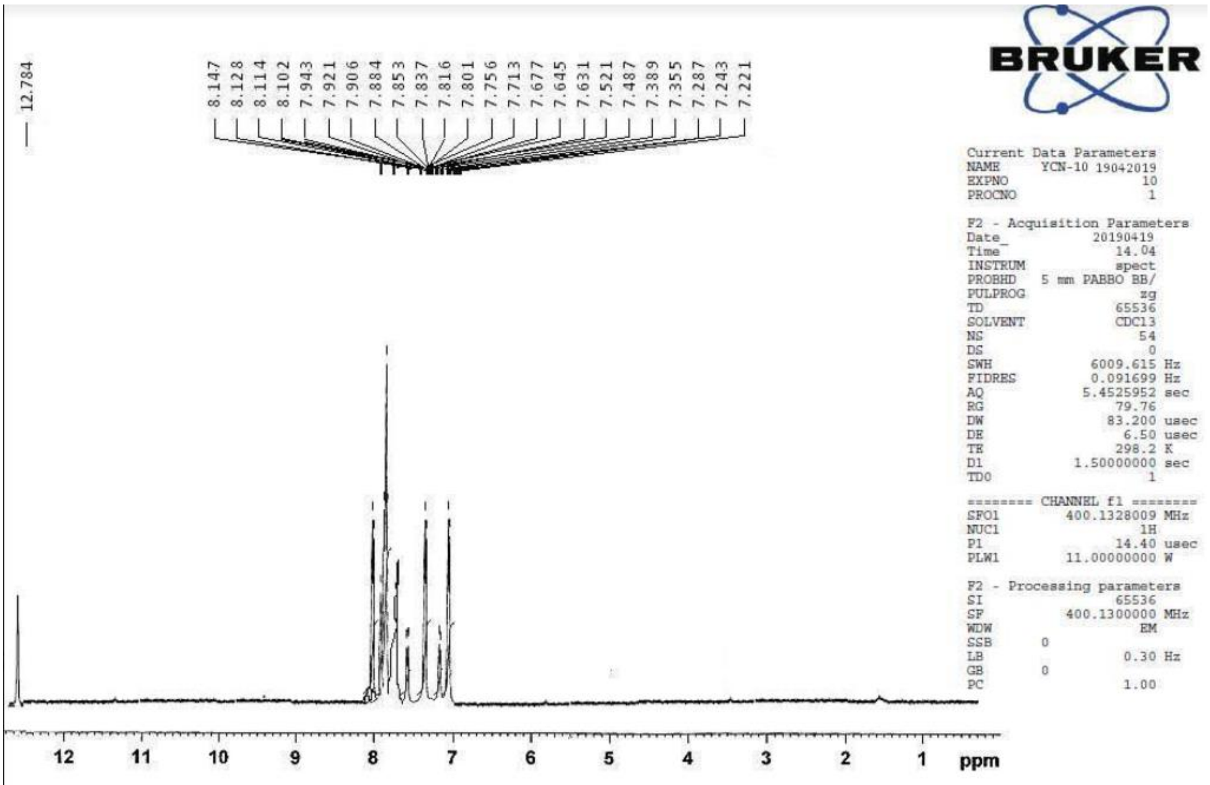

2-(3-Nitrophenyl)-1H-benzimidazole (3h):

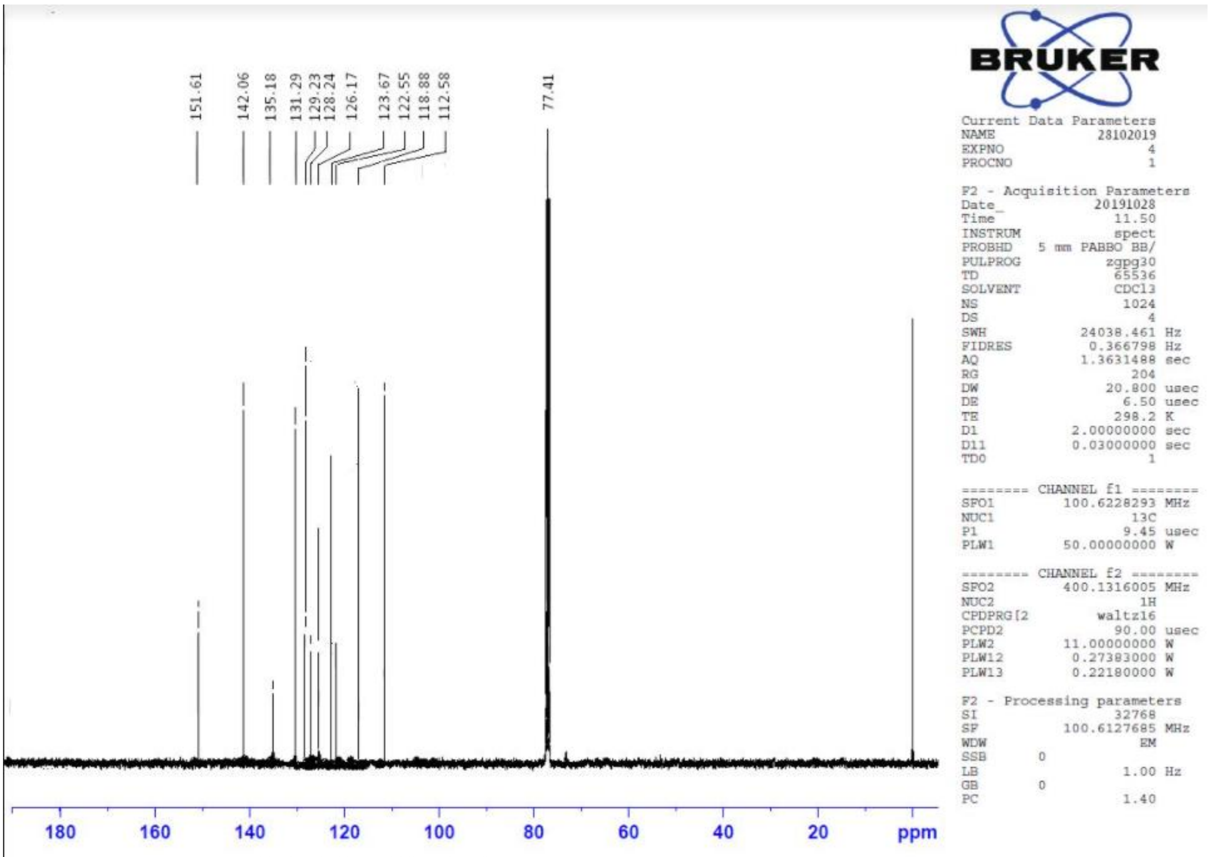

2-Phenyl-1H-benzimidazole (3a):

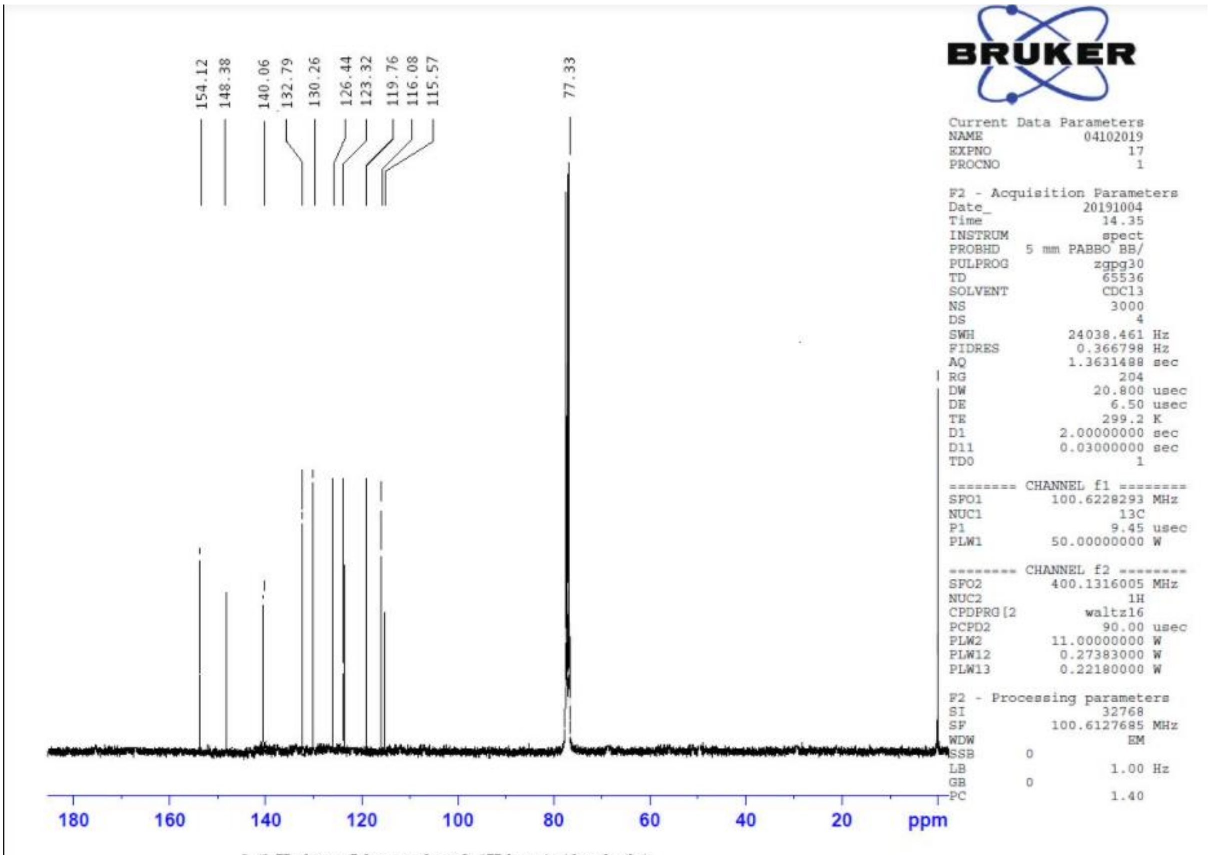

2-(2-Hydroxy-5-bromophenyl)-1H-benzimidazole (3g):

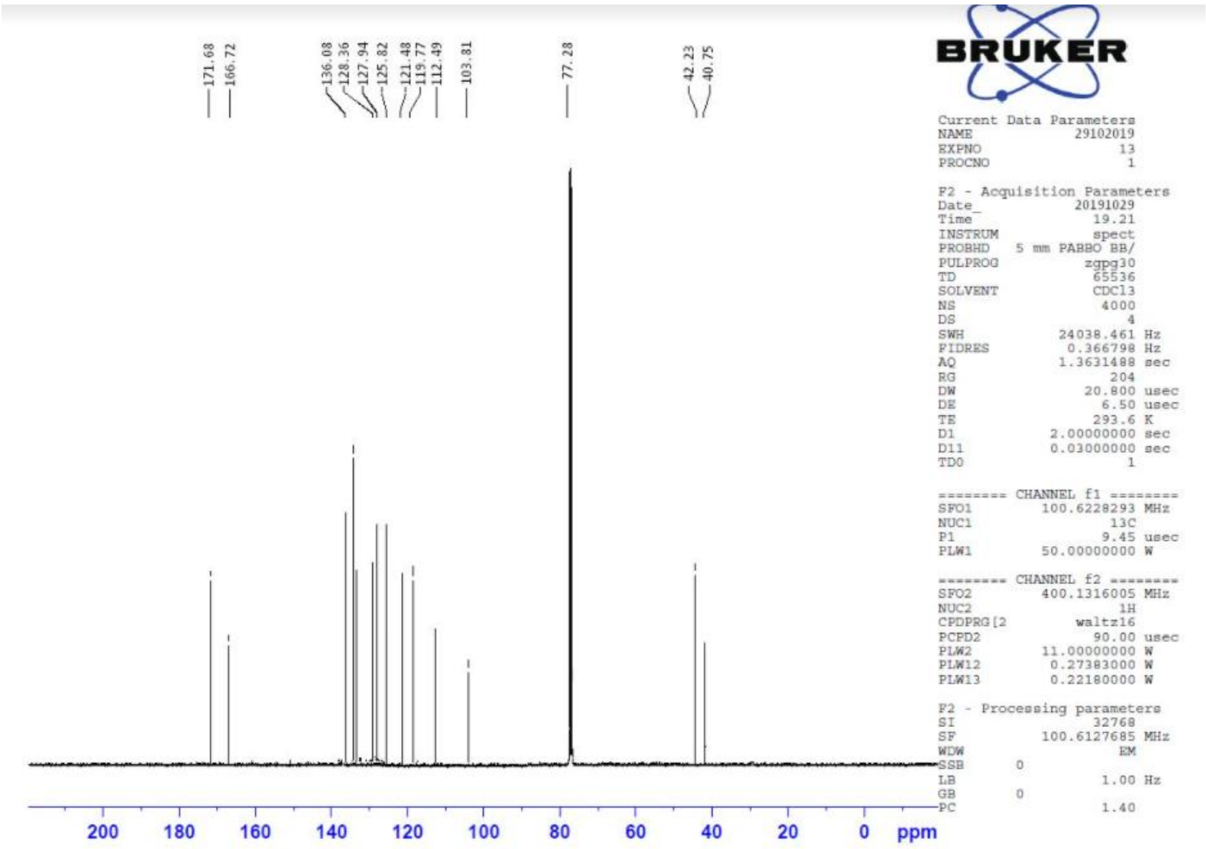

2-(4-methoxyphenyl)-1H-benzimidazole (3b):

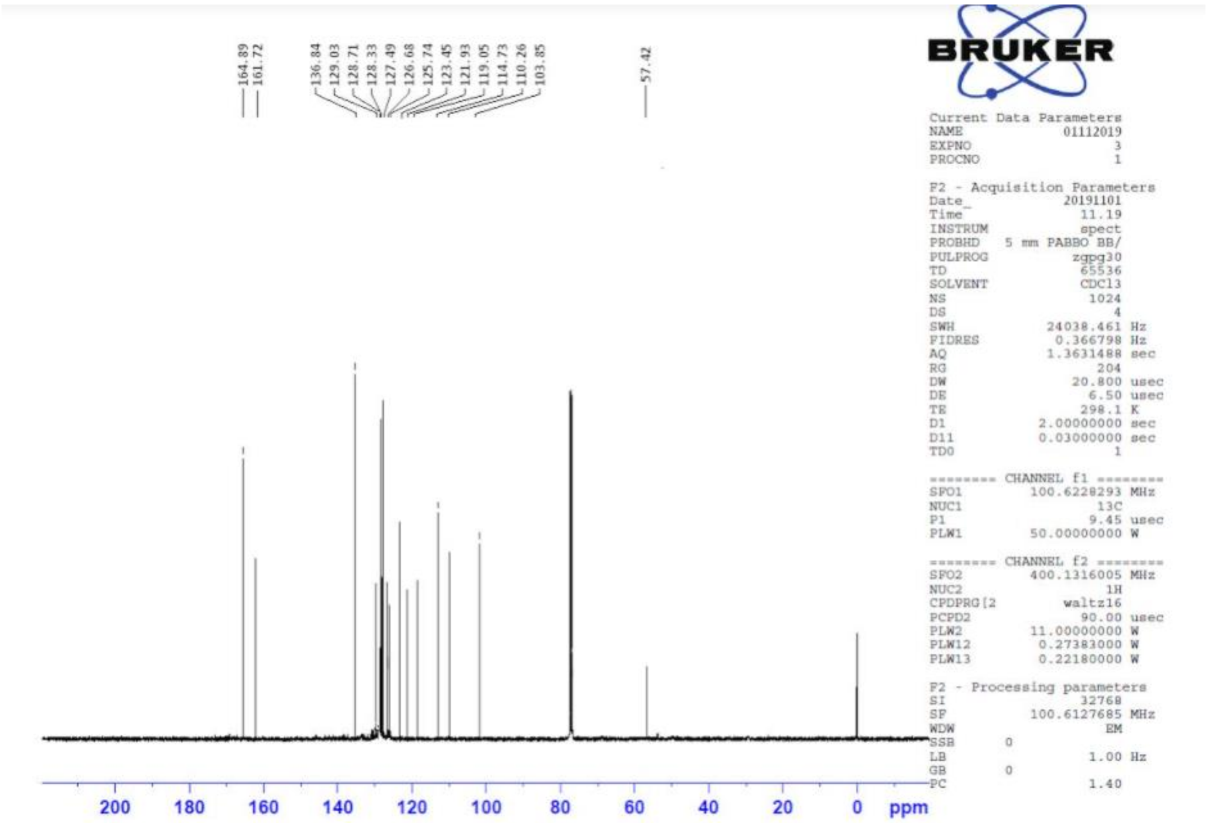

2-(3, 5-Dimethoxyphenylhenyl)-1H-benzimidazole

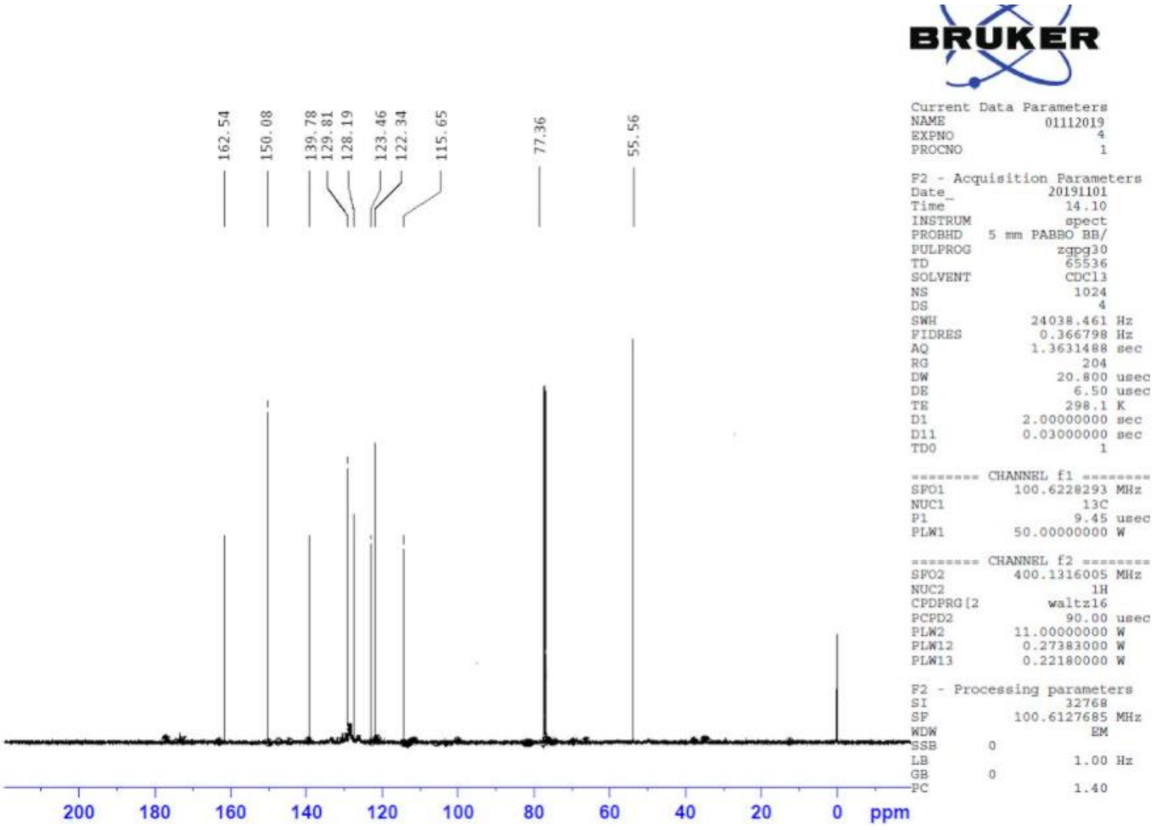

2-(4-methoxyphenyl)-1H-benzimidazole (3b):

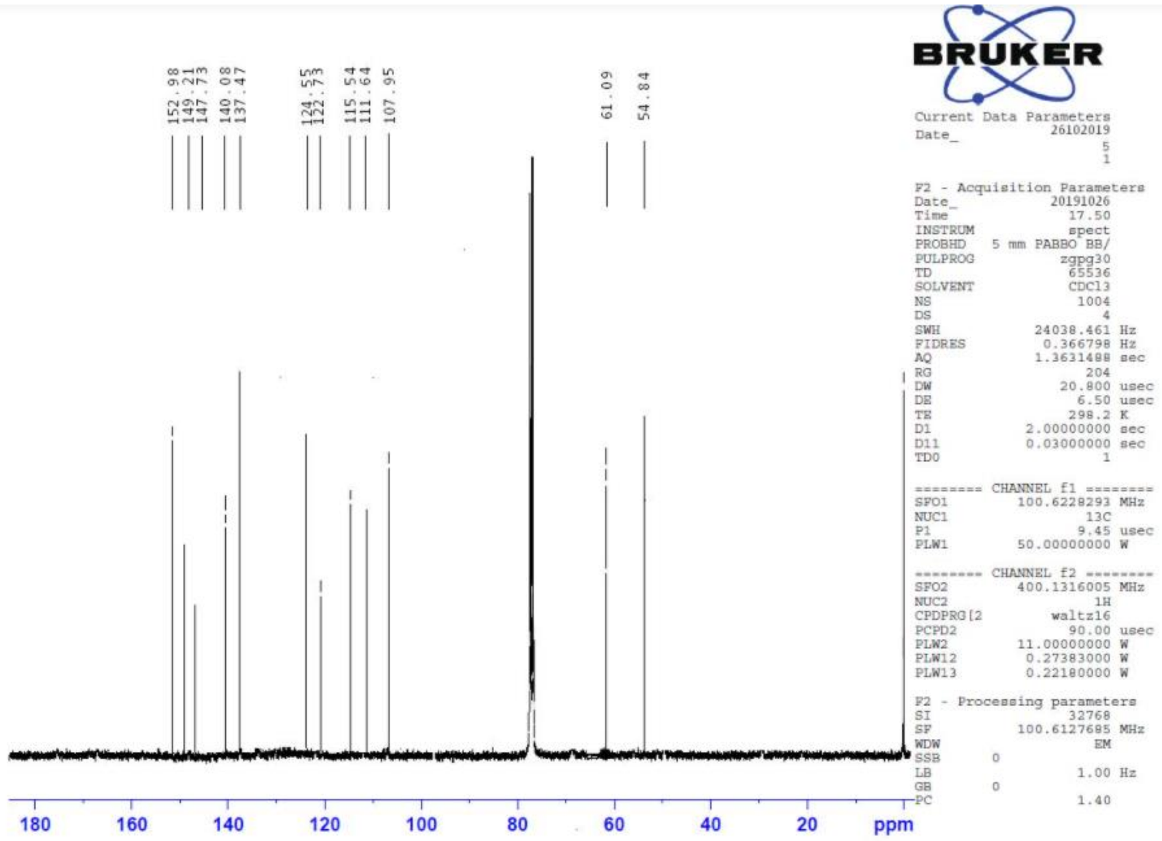

2-(2-bromo-3,4-dimethoxyphenyl)-1H-benzimidazole(3i):

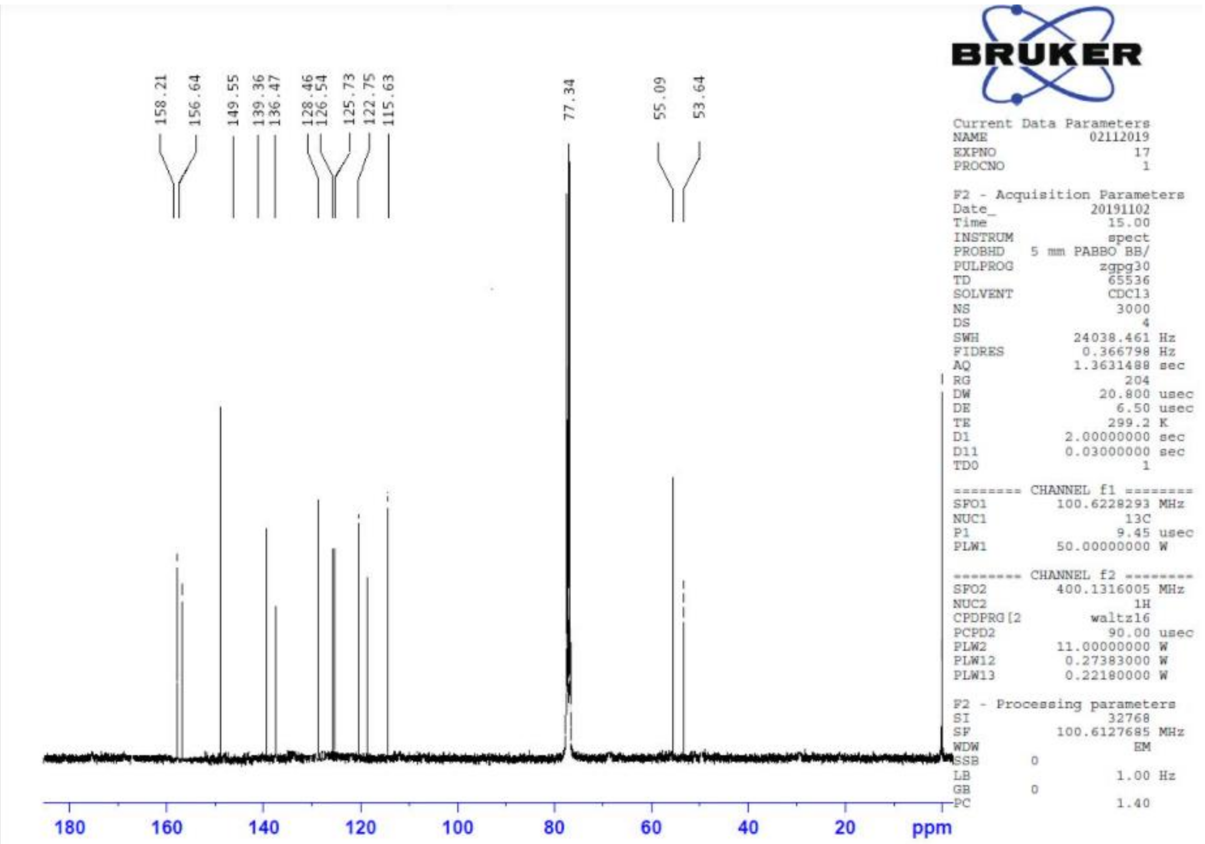

2-(2-iodo-3, 5-dimethoxyphenyl)-1H-benzimidazole (3j):
